# Blood Culture Utilization: How Many Follow-Up Cultures Are Needed?

**DOI:** 10.1017/ash.2025.312

**Published:** 2025-09-24

**Authors:** George Jones, Jennifer Hanrahan

**Affiliations:** 1 Eastern Virginia Medical School at Old Dominion University; 2 Eastern Virginia Medical School

## Abstract

**Background:** Follow-up blood cultures (BCx) are ordered after an initial positive culture in many instances. The number of follow-up cultures needed is not clear. Obtaining unnecessary BCx may cause unintended consequences. The optimal balance between stewardship and patient safety warrants investigation. We sought to assess the frequency with which a third set is positive after a negative second BCx. **Methods:** We conducted a retrospective study of BCx submitted to the microbiology laboratory from 1/1/18-11/1/23. We included all patients ≥18 years who had at least two follow-up BCx drawn 24-72 hours after an initial positive culture. Data were collected from electronic medical records. Cultures obtained within two hours of each other were counted as one set. Different strains of an organism were considered to be different organisms. Patients were divided into four groups based on BCx positivity, with a focus on the cohort with a positive culture after a negative follow-up set. **Results:** 28,875 patients had an initial positive BCx, of which 2,636 had at least two follow-up cultures drawn in the selected timeframe. Within this group, 585 (22.2%) had two positive follow-up sets, 1500 (56.9%) had two negative, 431 (16.4%) had a positive followed by a negative, and 120 (4.6%) had a negative followed by a positive. Of this cohort, 71 (2.7%) grew the same organism in the initial and second follow-up cultures, while 49 (1.9%) did not. In the same-organism subset, the most commonly identified bacteria were coagulase-negative staphylococci (n=21; 0.8%), gram-negative bacteria (n=17; 0.6%), methicillin-sensitive Staphylococcus aureus (n=13; 0.5%), and methicillin-resistant S. aureus (n=7; 0.3%). The most frequently isolated organisms in this subset were S. aureus (n=20; 0.8%), Staphylococcus epidermidis (n=16; 0.6%), and Escherichia coli (n=11; 0.4%). In the different-organism subgroup, 35 (1.3%) of the second follow-up sets had suspected contamination, though true bacteremia from skin/soft tissue (n=4; 0.2%), central line (n=4; 0.2%), unknown (n=3; 0.1%), and other sources was observed, often due to S. aureus (n=4; 0.2%), E. coli (n=2; 0.1%), and Candida (n=2; 0.1%). **Conclusion:** The number of patients with ongoing bacteremia that would have been missed with one follow-up BCx was small. The skip phenomenon has been described with S. aureus but was seen with gram-negatives as well. The second follow-up cultures were sometimes positive for contaminants. Further data are needed to determine when two follow-up sets should be obtained rather than one.

